# Effects of the SGLT2 inhibitor dapagliflozin on cardiac function evaluated by impedance cardiography in patients with type 2 diabetes. Secondary analysis of a randomized placebo-controlled trial

**DOI:** 10.1186/s12933-019-0910-5

**Published:** 2019-08-14

**Authors:** Benedetta Maria Bonora, Saula Vigili de Kreutzenberg, Angelo Avogaro, Gian Paolo Fadini

**Affiliations:** Department of Medicine, University of Padova, Via Giustiniani 2, 35128 Padua, Italy

**Keywords:** Type 2 diabetes, Sodium glucose cotransporter-2 inhibitor, Dapagliflozin, Heart failure, Impedance cardiography

## Abstract

**Background and aims:**

Cardiovascular outcome trials have documented a strong benefit of sodium glucose cotransporter-2 inhibitors (SGLT2i) on the risk of hospitalization for heart failure (HF) in patients with type 2 diabetes (T2D) with or without established cardiovascular disease or prior history of HF. The mechanisms, however, are not entirely clear. We aimed to evaluate whether treatment with SGLT2i affected cardiac function using impedance cardiography (ICG) in a randomized placebo-controlled trial.

**Materials and methods:**

Thirty-three patients with T2D were randomized to receive blind dapagliflozin 10 mg or matching placebo for 12-week on top of their ongoing glucose lowering medication regimen. Cardiac function was evaluated by resting ICG at baseline and at the end of the 12-week treatment period. ICG is a non-invasive technology based on the continuous measurement of thoracic electrical conductivity to process a cardiodynamic parameters related to fluid content, blood flow, cardiac function, and circulatory function. We also evaluated changes in glycaemic control, blood pressure, and body weight.

**Results:**

Thirty-one patients completed the study, 1 was excluded because ICG data was missing. Patients included in the final analysis were on average 63.4-year-old, with a known diabetes duration of 14.1 years and a baseline HbA1c of 8.2% (66 mmol/mol). 63.3% of patients had established cardiovascular disease (symptomatic or asymptomatic) and 36.7% had microangiopathy, but none had a prior history of HF. After 12 weeks, patients randomized to dapagliflozin, as compared to those randomized to placebo, showed improvements in HbA1c (− 1.2%; 13 mmol/mol), systolic blood pressure (− 3.7 mmHg), and body weight (− 3.3 kg). Based on ICG, in both groups, we detected no significant change in parameters of blood flow (stroke volume, cardiac output, cardiac index), systolic function (ejection fraction, acceleration and velocity indexes, systolic time ratio), circulatory function (systemic vascular resistance index), and fluid status (thoracic fluid content) after treatment.

**Conclusion:**

This is the first study exploring cardiac effects of SGLT2i using ICG in T2D. We observed no change in cardiac function parameters estimated by ICG in T2D patients who received dapagliflozin versus placebo for 12 weeks.

*Trial registration* ClinicalTrial.gov NCT02327039. Registered 30 December 2014

## Background

Patients with type 2 diabetes (T2D) have a two-to-fivefold increased risk of developing heart failure (HF) compare to non-diabetic patients [1]. This risk remains high despite control of known major cardiovascular risk factors [2]. HF is one of the first manifestations of T2D-related cardiovascular disease [3] and is one of the leading causes of hospitalizations in this population [4].

The presence of T2D negatively impacts HF outcomes. Patients with T2D are more likely to be hospitalized for HF [5], to be re-hospitalized for HF [6], and to experience a longer hospital stay [7] compared to patients without diabetes. Moreover, patients with T2D and HF have a higher risk of cardiovascular and all-cause mortality than HF patients without T2D [5].

Over the past decade, in patients with T2D, ischemic heart disease, and stroke mortality has declined, especially in men, but heart failure deaths did not change [8]. Therefore, therapeutic strategies able to reduce the HF risk are particularly appealing.

Sodium glucose co-transporter 2 inhibitors (SGLT2i) induce glycosuria, thereby reducing plasma glucose, blood pressure, and body weight [9]. The recent results of the four cardiovascular outcome trials (CVOTs) with SGLT2i have shown remarkable benefits in reducing HF hospitalization by about 30% in patients with T2D with or without established cardiovascular disease and also in patient with or without history of heart failure [10–13]. In addition, large retrospective studies confirmed that treatment with SGLT2i, when compared with other glucose-lowering medications (GLMs), was associated with lower rates of hospitalization for HF among patients with and without history of cardiovascular disease [14–16].

Several possible pathophysiological mechanisms for the protection exerted by SGLT2i on HF risk have been proposed [17], but not yet fully elucidated and mostly not confirmed in human studies.

In this study, we aimed to evaluate whether treatment with SGLT2i affected hemodynamic variables and cardiac function using impedance cardiography (ICG) in a randomized placebo-controlled trial.

ICG has been proposed as a non-invasive method of hemodynamic monitoring especially in critically ill and surgical patients [18]. ICG measures continuously the resistance (impedance) to the transmission of a low intensity electrical current through the chest. The changes in the resistance over time are proportional to the dynamic changes during each cardiac cycle. This allow the calculation of stroke volume (SV), cardiac output (CO) and other hemodynamic variables [19].

## Materials and methods

### Study design and participants

The study was approved by the Ethical Committee of the University Hospital of Padova and was conducted in accordance with the Declaration of Helsinki. All patients signed written informed consent. The trial was registered in ClinicalTrial.gov (NCT02327039). Patients were recruited between April 2015 and June 2016 at the diabetes outpatient clinic of the University Hospital of Padova. Eligible patients were randomly assigned (1:1) to one of 2 treatment groups, dapagliflozin 10 mg or matching placebo, on top of their GLMs for 12 weeks, based on a computer-generated sequence.

For safety reasons, the study was single blind meaning that patients, but not the clinical study staff, were unaware of the allocated treatment. However, the study staff in charge of the primary and secondary end-point evaluation was kept blind, thereby avoiding any interference on the study results. To guarantee concealment, pills and dispensers of dapagliflozin and placebo were identical.

Study details and the full eligibility criteria of the trial have been reported previously [20]. Briefly, inclusion criteria were: T2D patients aged 18 to 75 years, underlying therapy with oral GLMs and/or insulin. Major exclusion criteria were: acute illness or infection; recent surgery, trauma, or cardiovascular event; alcoholism; chronic liver disease; chronic kidney disease, defined as an estimated glomerular filtration rate (eGFR, calculated with CKD-EPI equation) < 60 mL/min/1.73 m^2^ [21]; HF New York Heart Association classes III to IV; history of hypotension or episodes of volume depletion/dehydration; previous history of recurrent urinary tract infections or genital infections; and pregnancy or lactation.

Diabetic retinopathy was defined based on digital fundus photography scored remotely by expert ophthalmologists and graded according to the Early Treatment of Diabetic Retinopathy Study (ETDRS) [22]. Somatic peripheral neuropathy was defined, after exclusion of non-diabetic causes, in the presence of typical sensory or motor symptoms (numbness, tingling, or pain in the toes, feet, legs, hands, arms, and fingers, or wasting of the muscles of the feet or hands), confirmed by clinical examination (ankle reflexes, vibratory perception threshold, pinprick, and 10-g monofilament sensitivity) and eventual determination of neural conduction velocity.

Coronary artery disease was defined as a history of myocardial infarction or angina, evidence of significant coronary artery disease at coronary angiography, or history of revascularization. History of HF was defined as a previous hospitalization for HF or clinical manifestations of HF. Echocardiographic abnormalities were evaluated according the current guidelines of American Society of Echocardiography/European Association of Cardiovascular Imaging [23, 24]. Systolic left ventricular (LV) dysfunction was defined as an ejection fraction < 40%. LV hypertrophy was defined by either an LV mass index of > 115 g/m^2^ for men and > 95 g/m^2^ for women indexed to body surface area. Diastolic LV disfunction was defined as recommended by the American Society of Echocardiography and the European Association of Cardiovascular Imaging [24, 25].

Peripheral arterial disease was defined as a history of claudication or rest pain, significant stenosis in leg arteries, or history of revascularization. Asymptomatic atherosclerosis was defined as the presence of carotid artery plaques (stenosis > 15%) at routine ultrasound examination.

The primary endpoint of the study was the change from baseline in cholesterol efflux capacity, which has been described previously [20]. ICG determination of cardiac function was an exploratory endpoint. At the beginning (before initiating randomized treatment) and at the end of the study (after 12 weeks of treatment) patients accessed the outpatient clinic and underwent a continuous ICG monitoring (Niccomo; Medis, Ilmenau, Germany) to record hemodynamic variables in a fasting condition for 20 min.

### ICG

The ICG methodology used in this study was similar to that used in previous clinical studies [26, 27]. ICG is a non-invasive method based on the assumption that changes in intra-thoracic blood volume during the cardiac cycle induce changes in the electrical conductivity and impedance of the thorax and that these changes are mainly related to changes in aortic volume. The changes in the thoracic impedance are detected by electrodes assessing the difference between the applied voltage and the detected voltage after applying a small electrical continuous current through the thorax. Four couples of electrodes were placed at the neck and thorax of each subject to detect variations in thoracic bioimpedance. The system was coupled with a standard sphygmomanometer transducer that measured blood pressure at given intervals.

Measuring electrocardiogram (ECG) together with ICG, the left ventricular ejection time (LVET) was directly measured [28]. LV SV was calculated using the formula described by Sramek and Bernstein [29]. Thoracic fluid content (TFC) was calculated as 1000/baseline impedance.

The Medis Niccomo ICG device processed these changes with a dedicated algorithm (physiologic adaptive signal analysis) coupling impedance signal and waveform with ECG, noninvasively and continuously provided the following hemodynamic variables: heart rate (HR); systolic blood pressure (SBP), diastolic blood pressure (DBP); SV, stroke index [SI (SV normalized for body surface area)]; CO (SV × HR); cardiac index [CI (CO normalized for body surface area)]; ejection fraction (EF); acceleration index [AI (the maximum acceleration of blood flow in the aorta during systole)]; velocity index [VI (the peak velocity of blood flow in the aorta during systole)]; systolic time ratio (STR); LVET [the time interval between the opening and the closing of the aortic valve (i.e., mechanical systole)]; left cardiac work (LCW) and LCW index (LCWI), which reflected the myocardial oxygen demand; systemic vascular resistance [SVR (estimate of “afterload)] and SVR index [SVRI (SVR normalised to body size); TFC (indicator of chest fluid status); TFC Index [TFCI (TFC, normalized for body surface area)]; total arterial compliance [TAC (indicator of the degree of peripheral arterial stiffness/compliance)], TAC index, [TACI (TAC normalised to body size)];

ICG variables were calculated by using the mean of 2-min beat-to-beat values for 20 min. After completion of the study protocol, original ICG recordings were downloaded in a personal computer for off-line analysis.

### Statistical analysis

Data are expressed as mean ± standard deviation if normal or as median (interquartile range) if not normal. Categorical variables are presented as numbers and percentages. Normality was checked using the Shapiro–Wilk test. Comparison between two groups were analyzed using the two-tailed unpaired Student’s *t* test for continuous variables and the χ^2^ test for categorical variables. For endpoints, we compared baseline with follow-up data using the two-tailed Wilcoxon rank test and calculated the change from baseline. Then, the changes from baseline in each group were compared using the two-tailed Mann–Whitney’s U test. Linear correlations were analyzed using the Pearson r coefficient. Statistical significance was accepted at p < 0.05 and SPSS version 22.0 was used.

## Results

**Fig. 1:**
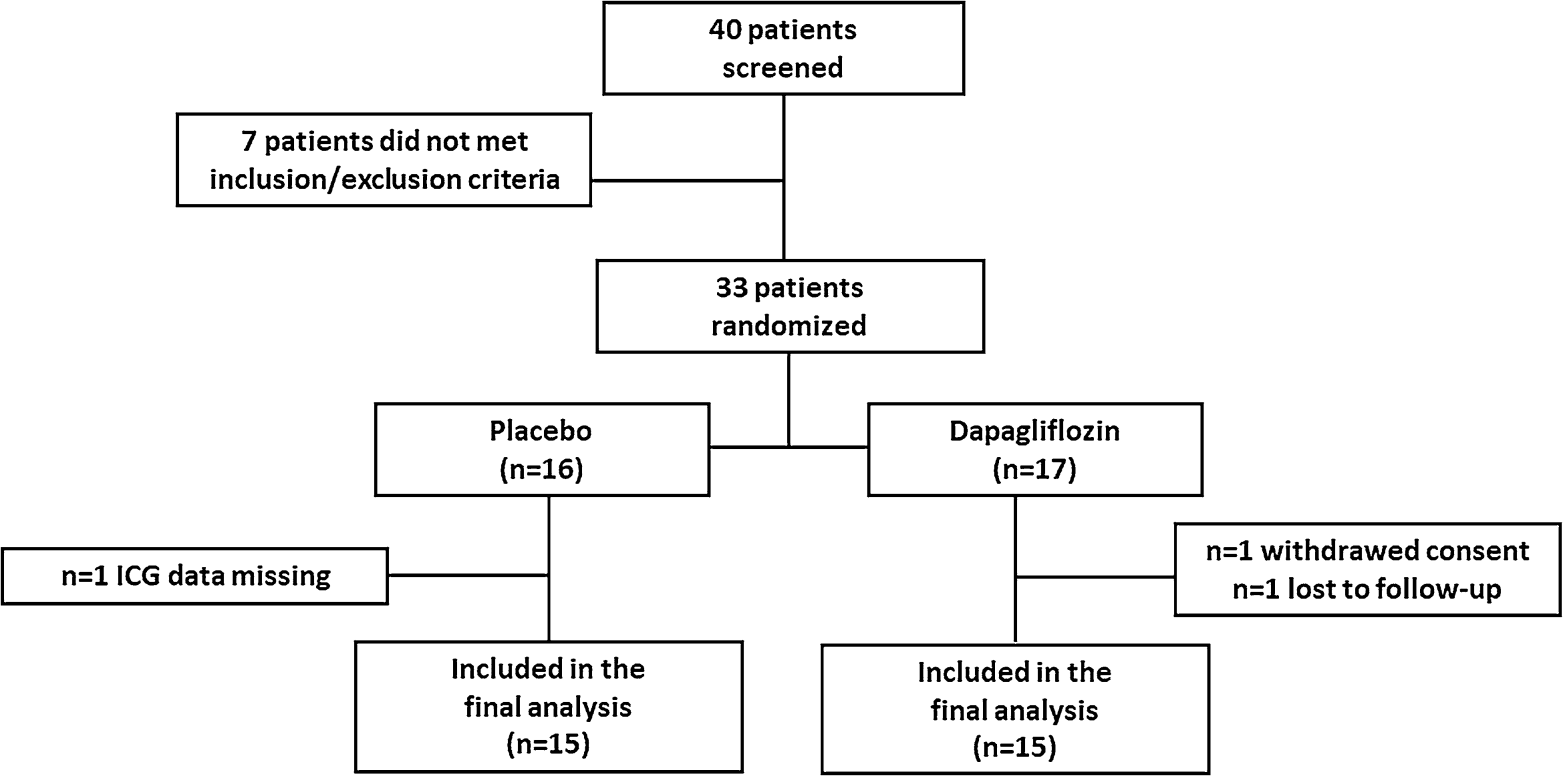
Study flow-chart with number of patients screened, randomized and included in the final analysis

Thirty-three patients were enrolled in the study. Seventeen were assigned to dapagliflozin 10 mg, 16 to matching placebo. Two patients in the dapagliflozin group were excluded because they withdraw consent or were lost to follow-up, one patient in the placebo group was excluded because ICG data were missing, leaving 30 completers for the analysis, 15 in each group (Fig. 1).

The baseline characteristics of the study population are summarized in Table 1. Patients were on average 63.7-year-old, with a known diabetes duration of 14.1 years and a baseline HbA1c of 8.2% (66 mmol/mol). The vast majority of patients (93.5%) were on metformin, 32.2% were on other oral GLMs, and 51.6% were concomitantly treated with insulin. 63.3% of patients had established cardiovascular disease (symptomatic or asymptomatic) and 35.4% had microangiopathy. No patient included in the study had a history of HF or a reduction of left ventricular ejection fraction. Almost half of the patients had LV hypertrophy (46.7%) and/or diastolic LV dysfunction (43.3%). After 12 weeks, dapagliflozin significantly reduced HbA1c by 0.9% and body weight by 3.1 kg. In the placebo group, HbA1c increased non-significantly by 0.4% and body weight remained stable (+ 0.2 kg).

**Table 1:** Baseline characteristic of study patients

Variable	All (n = 30)	Placebo (n = 15)	Dapagliflozin (n = 15)	*p*
Age, years	63.4 ± 6.9	61.0 ± 7.2	65.7 ± 5.9	0.057
Sex male, %	66.7	66.7	66.7	1.000
Body mass index, kg/m^2^	30.8 ± 5.2	32.8 ± 5.4	28.8 ± 4.3	0.032^§^
HbA1c, %	8.2 ± 0.8	8.2 ± 0.9	8.2 ± 0.6	0.981
Diabetes duration, years	14.1 ± 6.6	13.9 ± 5.2	14.2 ± 8.0	0.914
Hypertension, %	90.0	93.3	86.7	1.000
Smoking habit, %	16.7	20.0	13.3	1.000
Dyslipidemia, %	93.3	86.7	100	0.483
Creatinine, mg/dl	0.81 ± 0.2	0.81 ± 0.2	0.82 ± 0.2	0.921
eGFR (ml/min/1.73 m^2^)	90.6 ± 16.7	92.5 ± 16.9	88.7 ± 17.0	0.551
Retinopathy, %	26.7	33.3	20.0	0.682
Nephropathy, %	23.3	33.3	13.3	0.390
Neuropathy, %	3.3	6.7	0.0	1.000
Coronary artery disease, %	26.7	40.0	13.3	0.215
History of HF or systolic LV dysfunction, %	0.0	0.0	0.0	1.000
LV hypertrophy, %	46.7	53.3	40.0	0.715
Diastolic LV disfunction, %	43.3	46.7	40.0	1.000
Peripheral arterial disease, %	0.0	0.0	0.0	1.000
Cerebrovascular disease, %	56.7	53.3	60.0	1.000
Metformin, %	93.3	100	86.7	0.483
Sulphonylureas, %	20.0	20.0	20.0	1.000
DPP-4 inhibitors, %	20.0	13.3	26.7	0.651
Basal insulin, %	16.7	13.3	20.0	1.000
Basal-bolus insulin, %	36.7	46.7	26.7	0.500
ACE inhibitors/ARBs, %	83.3	86.7	80.0	1.000
Diuretics, %	33.3	26.7	40.0	0.700
Calcium channel blockers, %	30.0	33.3	26.7	1.000
Beta blocker, %	26.7	26.7	26.7	1.000
Statins, %	90.0	100.0	80.0	0.224
Anti-platelets, %	50.0	60.0	40.0	0.466

Data are expressed as mean ± standard, or percentages

^§^Not significant after correction of type I error

Compared with baseline, SBP declined by 4.7 ± 1.3 mmHg in the dapagliflozin group and by 1.0 ± 2.3 mmHg in the placebo group (p = 0.035). DBP declined by 1.3 ± 0.6 mmHg in the dapagliflozin group and by 0.4 ± 1.6 mmHg in the placebo group (p = 0.317) (Table 2).

**Table 2:** Fluid content, blood flow, cardiac function, and circulatory function at baseline and at study end in the two groups

Variable	Placebo (n = 15)				Dapagliflozin (n = 15)				p*
	Baseline	12 weeks	Change	p	Baseline	12 weeks	Change	p	
HR (beats/min)	68.6 (67.4; 78.3)	72.4 (66.9; 76.3)	− 0.9 (− 4.4; 5.0)	0.91	66.3 (61.1; 79.6)	69.6 (64.1; 81.2)	2.0 (− 3.1; 4.6)	0.39	0.53
SBP (mmHg)	129.0 (118.9; 135.6)	126.0 (118.1; 136.7)	2.47 (− 9.3; 6.0)	0.84	141.9 (133.4; 152.7)	136.3 (130.1; 147.3)	− 7.40 (− 8.9; 1.5)	0.03	0.04
DBP (mmHg)	76.1 (71.8; 81.4)	77.7 (73.9; 82.1)	2.3 (− 2.4; 4.6)	0.31	81.9 (75.4; 87.5)	82.0 (75.4; 84.7)	− 2.6 (− 3.8; 4.6)	0.91	0.32
SV (mL)	98.9 (72.3; 113.6)	85.7 (78.9; 100.8)	− 2.1 (− 16.6; 7.0)	0.33	94.8 (81.2; 104.0)	84.8 (70.3; 109.9)	− 7.8 (− 16.6; 14.4)	0.50	0.92
SI (mL/m^2^)	48.0 (36.7; 51.7)	42.9 (39.3; 44.7)	− 1.2 (− 8.2; 3.4)	0.43	47.1 (38.7; 56.3)	46.6 (39.2; 53.1)	− 3.3 (− 8.2; 7.4)	0.61	0.76
CO (L/min)	6.63 (5.42; 7.97)	6.31 (4.71; 7.98)	− 0.45 (− 0.79; 0.58)	0.46	6.94 (5.62; 7.81)	5.98 (5.52; 7.81)	− 0.49 (− 0.85; 1.06)	0.87	0.49
CI (L/min/m^2^)	3.33 (2.86; 3.62)	3.28 (2.47; 3.54)	− 0.21 (− 0.38; 0.29)	0.50	3.49 (2.86; 3.79)	3.45 (2.98; 3.66)	− 0.23 (− 0.37; 0.55)	0.82	0.52
EF (%)	65.6 (60.8; 69.0)	65.8 (62.3; 67.8)	0.6 (− 1.3; 4.4)	0.26	66.9 (61.5; 69.9)	65.6 (60.4; 69.6)	− 0.3 (− 4.4; 2.8)	0.43	0.19
AI (/100/s^2^)	47.6 (30.8; 53.6)	43.1 (36.1; 50.9)	− 0.1 (− 5.8; 4.2)	0.65	55.6 (39.8; 70.9)	58.6 (41.2; 65.4)	− 2.9 (− 10.2; 5.4)	0.39	0.85
VI (/1000/s)	33.1 (25.6; 42.4)	29.9 (25.7; 40.5)	− 4.1 (− 6.4; 2.4)	0.17	42.1 (35.4; 45.9)	43.6 (31.6; 49.0)	1.7 (− 3.0; 4.7)	0.69	0.18
STR	0.29 (0.24; 0.37)	0.28 (0.25; 0.34)	− 0.01 (− 0.07; 0.02)	0.22	0.27 (0.22; 0.35)	0.29 (0.23; 0.37)	0.00 (− 0.05; 0.07)	0.43	0.18
LVET (ms)	322.9 (311.7; 347.1)	330.0 (300.0; 364.8)	6.6 (− 10.4; 24.9)	0.57	337.0 (294.6; 376.6)	324.3 (298.8; 359.2)	1.8 (− 31.2; 17.4)	0.91	0.72
LCW (kg/min)	7.09 (6.18; 9.74)	6.71 (5.59; 9.50)	− 0.16 (− 0.48; 0.52)	0.89	9.30 (6.57; 9.82)	7.50 (6.54; 9.68)	0.44 (− 0.92; 1.05)	0.96	0.85
LCWI (kg × min × m^−2^)	3.66 (3.20; 4.37)	3.53 (2.90; 4.32)	− 0.08 (− 0.22; 0.26)	0.98	4.48 (3.37; 4.58)	4.12 (3.49; 4.93)	0.23 (− 0.45; 0.57)	0.82	0.77
SVR (dyne × s × cm^−5^)	924.0 (878.9; 1326.9)	1030.9 (904.3; 1374.6)	109.7 (− 76.6; 185.0)	0.36	1116.0 (965.8; 1358.8)	1157.3 (962.1; 1394.8)	81.1 (− 190.5; 174.4)	0.96	0.60
SVRI (dyne × s × cm^−5^ × m^−2^)	2051.9 (1770.6; 2581.4)	2119.0 (1887.7; 2672.8)	151.7 (− 150.86; 391.7)	0.39	2162.1 (1953.4; 2494.4)	2037.5 (1971.8; 2557.1)	150.7 (− 437.4; 310.5)	0.61	0.58
TFC (k/Ohm)	33.4 (29.0; 37.3)	29.9 (27.7; 34.9)	− 0.86 (− 3.5; 0.6)	0.16	31.4 (28.9; 35.1)	31.6 (29.4; 36.3)	− 0.8 (− 1.6; 1.6)	0.82	0.47
TFCI (k/Ohm/m^2^)	15.2 (13.5; 17.9)	14.5 (12.8; 17.7)	− 0.3 (− 1.0; 0.2)	0.28	15.7 (14.9; 17.6)	15.6 (14.8; 18.1)	0.0 (− 0.5; 0.4)	0.86	0.35
TAC (mL/mmHg)	2.19 (1.65; 2.31)	1.83 (1.38; 2.65)	0.00 (− 0.39; 0.29)	0.70	1.95 (1.2; 2.22)	1.60 (1.19; 2.22)	0.00 (− 0.20; 0.19)	1.00	0.95
TACI (mL/mmHg/m^2^)	1.03 (0.78; 1.07)	0.91 (0.71; 1.17)	0.00 (− 0.18; 0.16)	0.84	0.97 (0.72; 1.06)	0.80 (0.68; 1.07)	0.00 (− 0.09; 0.11)	0.88	0.97

Data are expressed as median (interquartile range)

* p-values of comparison between changes observed in dapagliflozin group versus those in placebo group

At baseline, there was no significant difference in ICG parameters between the two groups. In both groups, comparing data recorded at end of treatment with dapagliflozin or placebo to those recorded at baseline, we detected no significant change in parameters of blood flow (stroke volume, stroke index, cardiac output, cardiac index), systolic function (ejection fraction, acceleration and velocity indexes, systolic time ratio, left ventricular ejection time), circulatory function (systemic vascular resistance and systemic vascular resistance index, total arterial compliance and total arterial compliance index), and fluid status (thoracic fluid content and thoracic fluid content index) (Table 2). ICG-derived STR has been recently validated as a marker of LV diastolic dysfunction defined using echocardiography, with an accuracy of 97% [30]. At baseline, diastolic dysfunction based on the STR cut-off of 0.31 was present in 63.3% of patients (66.7% in the dapagliflozin group and 60% in the placebo group). At follow-up, the prevalence of diastolic dysfunction based on STR declined by 6.7% in the dapagliflozin group and remained stable in the placebo group (p = 0.325).

No significant correlation was detected between change in HbA1c or body weight from baseline to the end of follow-up and change in ICG parameters (not shown). In the whole study cohort, TFC correlated directly with total body water evaluated by bioelectrical impedance analysis (BIA) (r = 0.49; p < 0.001), but the change in TFC was not correlated to the change in total body water (r = 0.14; p = 0.456).

## Discussion

In this study, we found no significant effect of the SGLT2i dapagliflozin on measures of cardiac function evaluated by ICG. Since publication of the groundbreaking results of the EMPA-REG Outcome trial, the scientific community is struggling to understand the biological basis and the physiological mechanisms responsible for the cardiovascular benefits of SGLT2i, especially on HF.

Several possible pathophysiological mechanisms for the cardiovascular protection exerted by SGLT2i have been suggested, yet not formally proved. Briefly, these include reduction in preload, secondary to natriuresis and osmotic diuresis [31] and in afterload, secondary to reduction in blood pressure [32], myocardial Na^+^/H^+^ exchange inhibition [33, 34], reduction of necrosis and cardiac fibrosis [35, 36] and consequently of the development of HF. Reduction of inflammation and oxidative stress [37], restoration of the balance between pro- and anti-inflammatory adipokines and cytokine [38], reduction of epicardial adipose tissue mass [39] have been proposed as additional mechanisms. Moreover, the use of SGLT2i through an increased metabolism of free fatty acid and an increased production of ketone bodies may provide a more efficient source of energy for the myocardium [40].

Other possible mechanisms for the pleiotropic effects of SGLT2i on cardiovascular benefit involving the reduction of serum uric acid levels [41], haemoconcentration [42], improving endothelial function and aortic stiffness [43, 44] and may induce vasodilatation through activation of protein kinase G and the voltage-dependent K^+^ channel [45].

Some of these hypotheses result from studies in experimental models [33–37, 45] and should be confirmed in clinical studies, while other ones are just speculations [40] and require further research.

Despite all these mechanisms could contribute to protection against HF, information with respect to the direct effects of SGLT2i on myocardial function in humans is very limited. A small observational study showed that in patients with T2D and established cardiovascular disease, a short-term empagliflozin treatment was associated with a significant reduction in LV mass index and improved diastolic function measured by 2D echocardiography. No differences were found in LV systolic function, LV end diastolic volume, and LV end systolic volume [46]. Another observational study conducted in 37 patients with T2D with or without cardiovascular disease, showed that a treatment with canagliflozin improved LV diastolic function (E/e′ ratio) [47]. Similar results were found in a prospective observational study involving T2D with stable HF treated for 6 months with dapagliflozin [48]. However, these observational findings need to be confirmed in randomized controlled trials (RCTs). RCTs are currently being conducted to investigate the effects of SGLT2i on LV remodelling in patients with T2D and HF [49, 50]. Furthermore, large randomized-controlled trials investigating SGLT2i as a treatment for HF are ongoing and will enroll HF patients either with or without T2D, with reduced (DEFINE-HF, ClinicalTrial.gov registration no. NCT02653482) or preserved EF (PRESERVED-HF, ClinicalTrial.gov registration no. NCT03030235).

To the best of our knowledge, this is the first study exploring the cardiac effects of SGLT2i using ICG in T2D. This study suggests that, in T2D patients without a history of HF, a 12-week treatment with dapagliflozin exerted no overt effect on haemodynamic and cardiac function parameters compared to placebo. Power calculated a posteriori revealed that we could rule out an effect of dapagliflozin greater than 80 ms for LVET, 18 mL for SV (measures of systolic function) and 0.07 for STR.

Our data do not deny the strong protection provided by SGLT2i against the risk of HF, as demonstrated in solid and large CVOTs [10–13]. Rather, these negative findings may be attributed to several reasons that are at least in part related to study limitations. First, the observation time may be too short to detect changes in ICG parameters. However, in the 4 CVOTs with SGLT2i, the onset of the benefit in terms of reduction in HF hospitalization was very rapid. The curves started to separate in the first weeks after randomization, suggesting that diuretic or haemodynamic actions of SGLT2i could be at least in part responsible for the observed benefit.

Second, sample size may be too small to detect differences in ICG measures between the two groups of treatment. In fact, our study was designed to identify a significant change in the primary end-point, the effects of dapagliflozin compared to placebo on cholesterol efflux capacity [20] and power calculation was not performed based on ICG measurement variability. Thus, this exploratory re-analysis may be underpowered to detect significant changes in ICG parameters. Yet, in the same study, the analysis of body composition based on BIA clearly showed different effects of dapagliflozin versus placebo [20]. Therefore, we expected that the same sample size was sufficient to reveal overt effects of dapagliflozin on ICG, which is also based on impedance analysis. We detected a direct correlation between total body water and TFC, suggesting concordance of the two methods. Interestingly, while dapagliflozin reduced total body water by about 2.5 L [20], we herein found no change in TFC, suggesting that volume reduction occurred mainly in extra-thoracic compartments.

Furthermore, we recorded no parameter directly related to diastolic function. Although generally considered a measure of contractility, STR (the ratio between electric and mechanical systole) has a high accuracy for the diagnosis of diastolic dysfunction [30]. We found no effects of dapagliflozin on STR, either continuous or categorized according to the recommended cut-off [30], but we acknowledge that other parameters of diastolic function should be evaluated. Previous observational studies documented effects of SGLT2i on diastolic cardiac function in T2D, but a recent re-analysis of the DECLARE-TIMI58 study reported that dapagliflozin prevented HF in patients with a normal or reduced EF and improved survival especially in patients with reduced EF [51]. Thus, a selective effect of SGLT2i on diastolic function is unlikely to explain the observed benefit on HF rates.

Finally, the use of ICG for evaluation of cardiac variables has provided conflicting results in terms of reliability: studies comparing ICG with other methods to measure cardiac and hemodynamic parameters showed an agreement ranging from good [52–54] to poor [55, 56]. In addition, it should be mentioned that ICG is based on thoracic impedance triggering on cardiac cycle, from which all other measures are derived. Since SGLT2i typically reduce body fluid [57], it is also possible that the resulting change in impedance, that we have already demonstrated with BIA [20], masked the effects on cardiac function and hemodynamic parameters.

## Conclusions

Despite strong evidence that SGLT2i prevent HF in T2D with or without prior HF episodes, we found no effects of dapagliflozin on parameters of cardiac function measured by ICG. Short duration of treatment, small sample size, lack or specific diastolic function measures, limited correlation of ICG parameters with gold standard examination of cardiac function, and the masking diuretic effects of SGLT2i are all possible explanations of these negative results. In the end, we also speculate that SGLT2i exert no effects on cardiac function parameters in patients with normal cardiac function but may play a major role at the very early onset of cardiac disfunction, preventing hospitalization and mortality. Further studies are needed to address this hypothesis.

## Data Availability

Original data used to generate the present analysis can be obtained from the corresponding author upon reasonable request.

## Abbreviations

AI: acceleration index
ARB: angiotensin receptor blockers
BIA: bioelectrical impedance analysis
CI: cardiac index
CO: cardiac output
CVOTs: cardiovascular outcome trials
DBP: diastolic blood pressure
DPP4: dipeptidyl peptidase-4
ACE: angiotensin-converting enzyme
EF: ejection fraction
eGFR: estimated glomerular filtration rate
GLMs: glucose-lowering medications
HF: heart failure
HR: heart rate
ICG: impedance cardiography
LCW: left cardiac work
LCWI: left cardiac work index
LV: left ventricular
LVET: left ventricular ejection time
RCTs: randomized-controlled trials
SBP: systolic blood pressure
SGLT2i: sodium glucose cotransporter-2 inhibitors
SI: stroke index
STR: systolic time ratio
SV: stroke volume
SVR: systemic vascular resistance
SVRI: systemic vascular resistance
T2D: type 2 diabetes
TAC: total arterial compliance
TACI: total arterial compliance index
TFC: thoracic fluid content
TFCI: thoracic fluid content index
VI: velocity index
